# Spatially Varying Relationships between Alien Plant Distributions and Environmental Factors in South Korea

**DOI:** 10.3390/plants10071377

**Published:** 2021-07-05

**Authors:** Jeong-Soo Park, Hyohyemi Lee, Donghui Choi, Youngha Kim

**Affiliations:** Division of Ecological Safety, National Institute of Ecology, Seocheon 33657, Korea; hyohyemi@gmail.com (H.L.); dhchoi82@nie.re.kr (D.C.); khatru@nie.re.kr (Y.K.)

**Keywords:** invasive alien plant, geographically weighted regression, partial least squares regression, anthropogenic effect, *Lactuca scariola*, *Aster pilosus*

## Abstract

Invasive alien plants can severely threaten biodiversity and cause economic losses in the agricultural industry; therefore, identifying the critical environmental factors related to the distribution of alien plants plays a crucial role in ecosystem management. In this study, we applied partial least squares regression (PLSR) and geographically weighted regression (GWR) to estimate the important environmental factors affecting the spread of two invasive and expansive plants, *Lactuca scariola* L. and *Aster pilosus* Willd., across South Korea. GWR provides more accurate predictions than ordinary least squares regression, and the local coefficients of GWR allow for the determination of the spatial relationships between alien plant distributions and environmental variables. Based on the model’s results, the distributions of these alien species were significantly associated with anthropogenic effects, such as human population density, residential area, and road density. Furthermore, the two alien species can establish themselves in habitats where native plants cannot thrive, owing to their broad tolerance to temperature and drought conditions. This study suggests that urban development and expansion can facilitate the invasion of these species in metropolitan cities.

## 1. Introduction

Increasing global trade is one of the main reasons for biological invasions worldwide. Such invasions have become a matter of great concern because of their potential negative impacts on ecosystems and the agricultural industry [1]. Information regarding the critical environmental factors affecting the expansion of alien plants is required for many aspects of ecological research and for habitat protection strategies for native plant conservation [2,3]. The spread of invasive plants is determined by the complex interactions of diverse factors including their biological traits, land use, climate, and anthropogenic disturbances [4,5].

Numerous studies have been conducted on the relationships between alien plant distributions and environmental variables [6,7,8]. In this study, we applied geographically weighted regression (GWR), which can incorporate spatial non-stationarity and estimate local coefficients. Traditional statistical methods cannot accurately incorporate spatial non-stationarity, although environmental variables usually show strong spatial dependence (i.e., autocorrelation). Specifically, ordinary least squares (OLS) regression and the stationary coefficient model assume that the relationships between variables are the same across the entire space and compute the parameters as average values across all locations [9]. However, GWR can calculate the local parameters for different spatial regions which take neighboring observations into account [10]. GWR is a tool that is more frequently used in healthcare and demographic research rather than ecological studies [11,12,13].

Two invasive alien plants, *Aster pilosus* Willd. and *Lactuca scariola* L., were introduced into South Korea approximately 50 years ago [14,15]. These plants have rapidly spread across South Korea. This has been attributed to their tolerance of harsh environmental conditions and strong wind dispersal abilities [16,17,18]. Both species are designated as harmful invasive plants by the Ministry of the Environment of the Republic of Korea because of their negative impacts on ecosystem diversity. They are also considered troublesome weeds in several regions such as the United States, Europe, and Australia [17,18]. It is important to estimate the critical environmental factors that facilitate the spread and establishment of harmful alien plants. In particular, South Korea is likely to be increasingly prone to alien plant invasions owing to the increasing volume of trade and anthropogenic pressure on natural habitats.

The specific objectives of this study were to (1) estimate the effect of 13 environmental variables (human population density, mountain area, elevation, temperature, etc.) on alien plant distribution patterns using partial least squares regression; (2) explore the spatial non-stationarity of local coefficients using GWR; (3) delineate the risk areas where invasive alien plants could spread most easily.

## 2. Results

**Figure 1:**
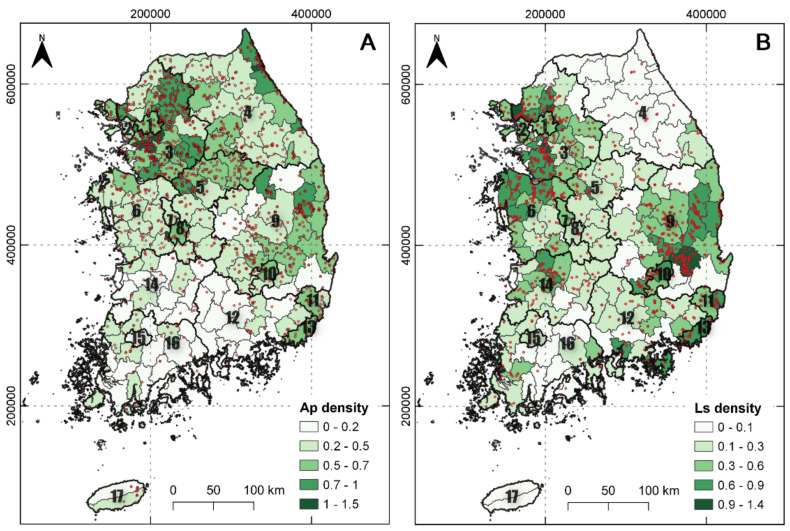
The density of *A. pilosus* (Ap) (**A**) and *L. scariola* (Ls) (**B**) in 165 districts (small polygons) in South Korea. The bold numbers in the polygons represent the 17 provinces of South Korea. The red points represent the points at which the two plant species were detected. The legends show log-transformed *A. pilosus* and *L. scariola* population density.

The spatial distributions of the two alien plant species are shown in Figure 1. *A. pilosus* was identified at 1700 geographical points throughout South Korea and *L. scariola* at 1253 points. Among the 17 provinces, the two alien plant populations were more frequently found in provinces 1, 2, 3, and 9 than in other provinces. Moreover, the southern districts had lower alien plant densities. The population densities of the two species exhibited large variability among the 165 districts. All the measured variables showed significant spatial autocorrelation, meaning that there was a tendency for districts that are close together in geographical space to have similar values. The *A. pilosus* population had a higher population density and stronger spatial autocorrelation than the *L. scariola* population.

**Figure 2:**
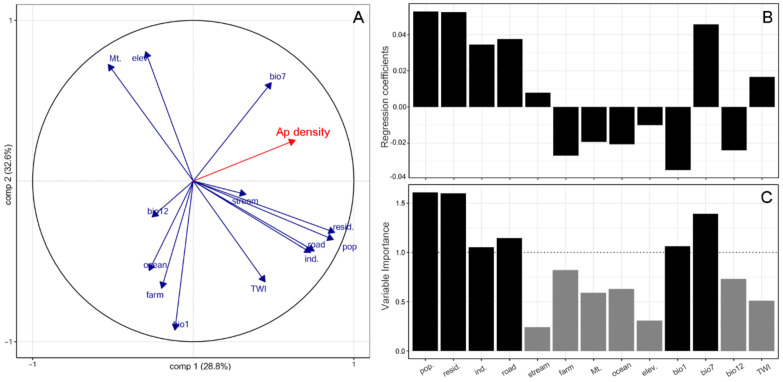
Partial least squares regression (PLSR) biplot of the first two components (**A**). Regression coefficient (**B**) and variable importance to the projection (VIP) (**C**) from the PLSR of *A. pilosus* (Ap) density. The variables with VIP > 1 are good candidates to explain *A. pilosus* density; pop.: human population density, resid.: residential area, ind.: industrial area, road: road length ratio, stream: stream area, farm: farm area, Mt.: mountain area, ocean: facing the ocean, elev.: elevation, bio1: annual mean temperature, bio7: annual temperature range, bio12: annual precipitation, and TWI: topographic wetness index.

The PLSR model showed that 13 variables influenced the densities of the two alien plant species differently. *A. pilosus*’ density was mainly explained in the PLSR model by the first two components (61.4%). Four explanatory variables (i.e., population density, residential area, road, and industrial area) were highly correlated with each other, which was also observed in the correlation matrix (Figure S1). The most influential predictor of *A. pilosus*’ density was identified as anthropogenic activity (i.e., population density, residential area, and road), followed by temperature variables (i.e., bio1 and bio7), based on the variable importance of the PLSR projection. Stream, farm, mountain, and the precipitation-related variables were only weakly influential compared to the other variables. Six explanatory variables (e.g., farm, mountain, ocean, elevation, and bio12) showed negative associations with *A. pilosus*’ density (Figure 2).

**Figure 3:**
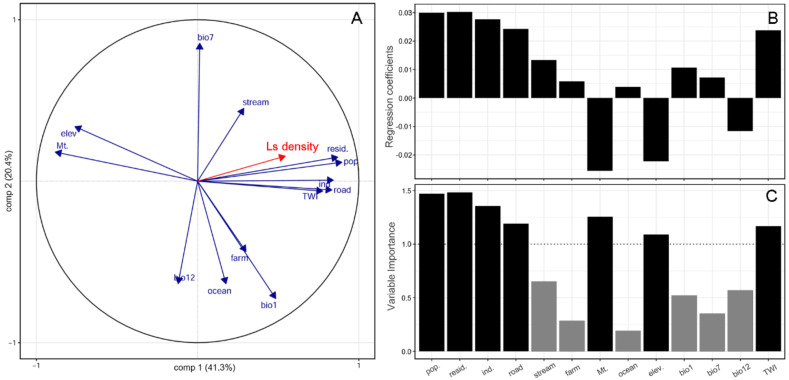
Partial least squares regression (PLSR) biplot of the first two components (**A**). Regression coefficient (**B**) and variable importance to the projection (VIP) (**C**) from the PLSR of *L. scariola* (Ls) density. The variables with VIP > 1 are good candidates to explain *L. scariola* density. Variable abbreviations are given in Figure 2.

The anthropogenic activity (population density, residential area, and industrial area) were also the most influential explanatory variables for *L. scariola* density, followed by mountain and the topographic wetness index (TWI). The regression coefficient of the PLSR showed that the mountain, elevation, and bio12 (annual precipitation) variables had negative associations with *L. scariola* density (Figure 3). The mountain, elevation, and TWI variables were significantly related to each other (Figure S2).

When the same data were used in the OLS and GWR models, the estimated parameters and model performances differed in the final models. Both explanatory variables (Residential area and Bio7) were positively associated with *A. pilosus* density at the *p* < 0.001 level in the OLS model. In contrast, the GWR model estimated that over 99.9% of the local coefficients for residential area were positively associated with *A. pilosus* density, and 17% of the local coefficients for bio7 were negatively associated with *A. pilosus* density. The model performances increased in the GWR model. For example, the GWR model accounted for 66% of the differences in *A. pilosus* density among the 165 districts, which is 22% higher than the OLS model (Table 1). The AIC value was also lower in the GWR model than the comparative value in the OLS model, suggesting that the variables showed a better fit in the GWR model. The OLS model for *L. scariola* density showed that the mountain variable was negatively associated with *L. scariola* density at the *p* = 0.03 level. The GWR model revealed that 98% of the local coefficients for mountain were negatively associated with *L. scariola* density, indicating that *L. scariola* population density decreases in 98% of the districts with higher mountain percentages. The GWR model for *L. scariola* accounted for 48% of the variation in *L. scariola* population density, which is 21% higher than the OLS model. The lower AIC value of the GWR model also shows that model performance is higher in the GWR model than in the OLS model. The Moran’s I of the residuals for the OLS model was higher than that for the GWR model, indicating that residuals in the OLS model had a greater tendency toward clustering than those in the GWR model.

**Figure 4:**
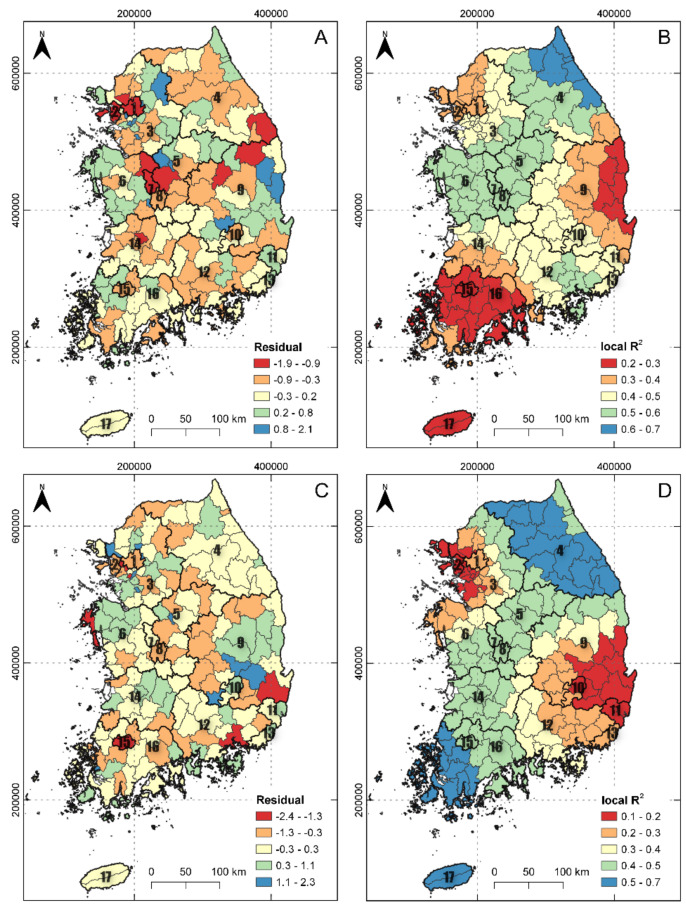
Spatial mapping of the residual and locally weighted coefficients of determination (*R^2^*) of *A. pilosus* density (**A**,**B**) and *L. scariola* density (**C**,**D**) in geographically weighted regression models.

Figure 4 shows the patterns of standardized residuals and local adjusted *R*^2^ values for the *A. pilosus* and *L. scariola* GWR models. The residual values in the GWR model were randomly distributed across the 165 districts. The *A. pilosus* model had lower residual values than the *L. scariola* model. The spatial distributions of the local *R*^2^ values show that lower values occurred in the east Gyeongbuk (9), Gwangju (15), Jeonnam (16), and Jeju (17) for the *A. pilosus* GWR model and in the Incheon (2), east Gyeongbuk (9), Daegu (10), and Ulsan (11) for the *L. scariola* GWR model. This indicates that the models did not fit well, and additional covariates are needed to explain the distributions of the two alien plants in those districts.

**Figure 5:**
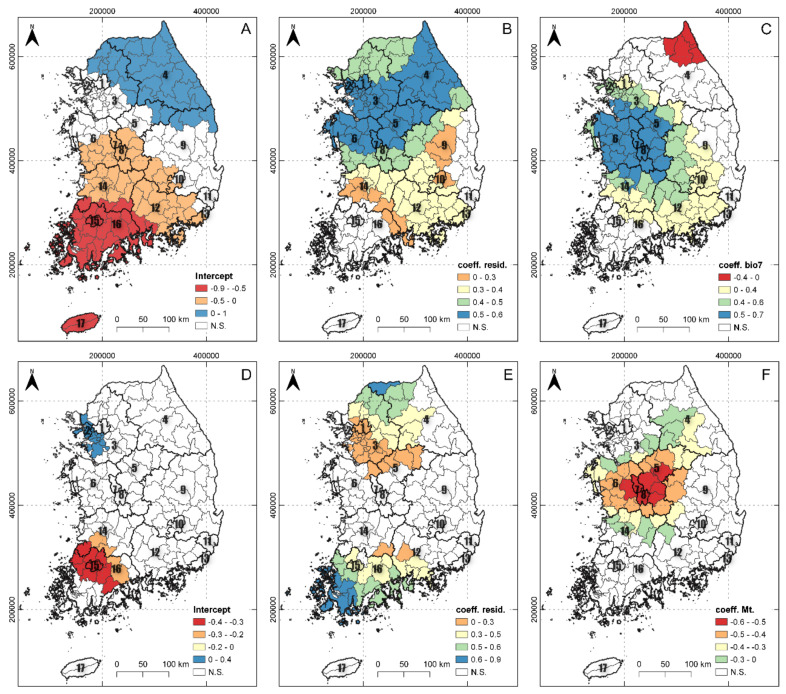
Spatial mapping of the coefficients of the *A. pilosus* GWR model (intercept (**A**), residential area (**B**), bio7 (**C**)) and *L. scariola* GWR model (intercept (**D**), residential area (**E**), mountain area (**F**)). The significance of the local coefficients was determined by pseudo t-values; the regions with *t*-values between −1.96 and +1.96 (nonsignificant parameters) are indicated in white; resid.: residential area, bio7: annual temperature range, and Mt.: mountain area.

The spatial variation in the parameter estimates for the local coefficients and *t*-values are shown in Figure 5. The map of the intercept term represents the distributions of the two alien plant species when the selected explanatory variables, such as residential area, bio7, and mountain, are equal to zero. Negative intercept values were found in southwest provinces. These spatial patterns imply that there were other influential variables that reduced alien plant distributions. The *A. pilosus* GWR model shows that the coefficients of residential area and bio7 were statistically significant for 131 and 100 of the 165 districts, respectively. Residential area showed positive effects in 131 districts, and the strongest effects were found in the northern districts. In addition, annual temperature range (bio7) showed positive effects in all districts, except for northeast Gangwon (4) Province. The *L. scariola* GWR model revealed that the coefficients of residential area and mountain were statistically significant in only 73 and 49 of the 165 districts, respectively, and that the two explanatory variables had positive effects on *L. scariola* distribution.

## 3. Discussion

This study applied GWR to explore the spatially non-stationary relationships between invasive alien plant distributions and environmental factors. Although OLS can show an intuitive global effect direction (either negative or positive), GWR provides more accurate predictions and a set of local coefficients, by reducing spatial autocorrelation [19,20]. Our results show that the distributions of the two alien plant species were spatially autocorrelated at this study scale. Furthermore, the main environmental variables that have a critical effect on alien plant distribution also showed strong spatial autocorrelation. We expect these GWR results to provide policy makers with a more specific basis for eradication and prevention strategies, because this model provides a local coefficient for each district.

Our study found faster geographical expansion and higher spatial clustering of the two alien plants in urbanized areas than in rural areas. Our results show that the invasiveness of the two alien plants was strongly associated with anthropogenic effects such as human population density, residential area, and road density. However, climate factors, such as annual mean temperature and precipitation, were not significantly associated with the densities of the two alien plants. We predict that these spatial distribution patterns are affected by seed dispersal strategy and human activity.

Previous studies reported that wind is the primary dispersal force for these species [17,18], owing to the morphological features of their seeds, such as the bristled pappi and lightweight achenes (less than 1 mg). This is similar to other Asteraceae plants [17,18,21]. Furthermore, both *A. pilosus* and *L. scariola* produce many seeds in autumn; approximately 25,000–58,000 and 10,000–100,000 seeds per plant, respectively [18,22]. In urban areas, high-rise buildings cause strong building winds, and fast-running vehicles generate strong winds along roadsides. Additionally, roads provide corridors through which seeds can be transported from one site to another via the mud attached to cars. The interconnected road systems between cities can therefore facilitate the expansion of alien Asteraceae plants [23,24].

Our study also revealed that the population densities of these species were higher in ruderal habitats than in natural habitats (i.e., mountain areas). The two studied alien plants frequently occur in disturbed ruderal areas such as roadsides, dumps, and fallow lands in Europe and North America [17,18]. *L. scariola* is considered an “r” strategist owing to its short life cycle, strong self-fertilization ability, and rapid germination and flowering [25]. Our results indicate that this species can germinate and successfully complete its life cycle in open ruderal habitats within a short period [16]. Temperature, photoperiod, and precipitation may be influential environmental factors for its flowering and fruiting [26]. Previous studies also mentioned that hot, dry weather in the summer may enhance fruiting in this species because low rainfall can maintain open habitats [22,24]. We suppose its seeds can be moved by wind more easily in open-flat sites than in mountainous regions.

*A. pilosus* commonly occurs in abandoned fields in the first year and becomes the dominant species in second- and third-year fields [18]. *A. pilosus* has various competitive strategies for establishment and expansion in disturbed ruderal habitats. First, under resource-limited conditions or in the winter season, this plant can survive in the form of rosettes, from which flowering stems can emerge the following summer [27]. Second, *Aster* frequently allocates a large proportion of its total biomass to stems and roots, which enhances its ability to acquire moisture and withstand considerable water limitation [18]. Lastly, high rates of photosynthesis were measured over a broad range of temperatures and under drought conditions, indicating that this plant is physiologically tolerant to harsh environmental conditions [27]. Our results also show that the population density of this plant was positively associated with annual temperature range (bio7), suggesting that this plant can adapt to a broad range of temperature conditions.

This study provides insight for policy makers to guide decisions regarding target locations and the effort necessary to control and prevent the spread of these alien plants. We predict that urban development and expansion may facilitate the invasion of these plants, especially around metropolitan cities (i.e., Seoul, Incheon, and Daegu). The annual alien plant, *L. scariola,* may be able to establish in urbanized areas more rapidly than perennial plants, but more effort may be needed to eradicate *A. pilosus* than *L. scariola.* Above all, natural habitats and forests should be protected from reckless development to prevent the expansion of these invasive alien plants.

## 4. Materials and Methods

### 4.1. Study Area

**Figure 6:**
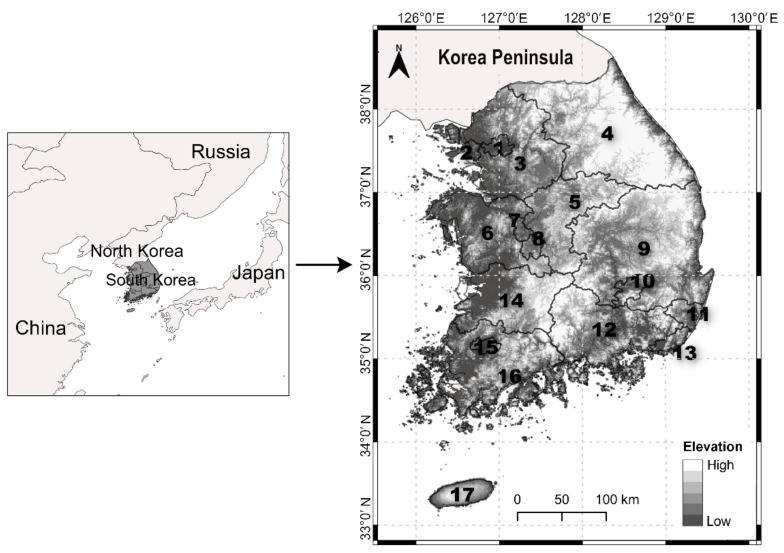
Location of the study area and the background topography. The bold numbers in the polygons represent the 17 provinces of South Korea.

A national non-native species survey was conducted all over South Korea to estimate the distribution status of alien species, which extends from 33°0′ to 38°9′ N and 124°5′ to 132°0′ E, covering an overall geographical area of nearly 100,340 km^2^ (Figure 6). Two districts (Ongjin-gun and Ulleung-gun), consisting of small islands, were excluded because of the difficult accessibility of the islands. Almost 64% of the land in South Korea is mountainous, followed by 16% which is agricultural land. The total population was 51.7 million people in 2020, and half of the population lived around Seoul and the capital cities in provinces 1, 2, and 3 [28]. According to the Köppen climate classification, the main climate types are a monsoon-influenced, hot-summer, humid continental climate (in the northern inland regions) and a humid, subtropical climate (in the southern coastal regions) [29]. The mean annual temperature is 12.5 °C, and the mean precipitation over the last 30 years is approximately 1300 mm [30].

### 4.2. Study Species

*Aster pilosus* Willd (syn: *Symphyotrichum pilosum*; frost aster) is an herbaceous perennial plant (Asteraceae). This plant is native to North America and was introduced into European countries (France, Germany, Spain, Belgium, Italy, etc.), India, and South Korea [31]. This species has expanded rapidly throughout South Korea since it was first recorded in the 1980s [15]. In their late autumn flowering season, the large robust individuals have a shrubby appearance. The Ministry of the Environment of the Republic of Korea designated this species as a harmful invasive plant in 2009 because of its invasiveness and the possibility that it decreases biodiversity. Frost aster achenes are mainly dispersed by wind to distant habitats from late fall to early winter and can also be propagated via division of the rosettes and from stem cuttings. The number of achenes produced per plant varies from 25,000 (first year) to 58,000 (second year) in the field. This plant overwinters as seeds or rosettes. After the flowering stems die down, the individuals can survive winter in the form of rosettes [18].

*Lactuca scariola* L. (prickly lettuce) is an herbaceous annual or biennial plant (Asteraceae). This plant is native to Eurasian and has spread globally, including East Asia, South Africa, North America, and Australia [31]. This plant was introduced into South Korea in the 1980s and has rapidly expanded across the country. *L. scariola* was also designated as a harmful invasive plant in 2012 owing to its competition with native plants and crops. This plant is a problematic weed in Australia and North America [32]. This plant can produce up to 87,000 seeds per plant in non-crop areas [33] and can germinate quickly under optimal temperature and soil moisture conditions.

### 4.3. Data Preparation

The distribution data for the alien plants were mainly obtained from national non-native species survey, which was conducted 165 districts of South Korea (total 167 districts) by 20 scientists from 2015 to 2019, and from the Clearing-House Mechanism (CHM) website of Korea (https://www.kbr.go.kr/home/bio. accessed on 8 January 2021). We selected 12 explanatory variables associated with alien plant expansion and settlement (Table 2). All explanatory variables were categorized into four groups based on their information properties and data sources: anthropogenic activity, land use, topographic, and climate properties. The anthropogenic activity and land-use variables were obtained from a public data portal [34]. We extracted elevation and TWI data from the digital elevation model (1 km × 1 km resolution) and current raster climate datasets (1980–2010) were downloaded from WorldClim (version 2.1) [35]. We selected three climate variables considering the eco-physiological characteristics of the two alien plants (i.e., annual mean air temperature (bio1), annual temperature range (bio7), and annual precipitation (bio12)). The obtained topographic and climatic variables were calculated as the average values of each district using QGIS (version 3.16). To determine the spatial non-stationarity of each variable, the highest values of Moran’s I statistic were obtained for the climatic and topographic properties. Skewed data sets (population density, residential area, industrial area, road, and stream) were log-transformed to obtain near-normal distributions, based on the Shapiro–Wilk test. Then, min–max normalization was performed to normalize all the variables. Pearson’s correlations (r pairwise ≥ 0.7) and variance inflation factors (VIF ≥ 3) were calculated to avoid potential multicollinearity among the explanatory variables [36].

### 4.4. Data Analysis

Geographically weighted regression (GWR) was applied to explore the local spatial relationships between alien plant distributions and explanatory variables. It is a useful regression method to examine geographically non-stationarity relationships and estimate the causal relationships between a response variable (y) and a set of explanatory variables ($x_{i}$) (i*=1, 2, …, n*) at a local scale [37]. GWR generates a separate coefficient for each location, which can be expressed as follows [38]:

(1) $$y_{i}=\beta_{0}\left(u_{i},v_{i}\right)+\sum \beta_{k}\left(u_{i},v_{i}\right)x_{ik}+\varepsilon_{i},$$

where $y_{i}$ is the response variable, $\beta_{k}$ is the coefficient, $x_{ik}$ is the explanatory variable, $\left(u_{i},v_{i}\right)$ are the coordinates of i, and $\varepsilon_{i}$ is the error term; $\varepsilon_{i}\sim N\left(0,\sigma^{2}\right)$.

The coefficient for this model takes the form of:

(2) $${\hat{\beta }}_{i}={\left[X^{T}W\left(u_{i},v_{i}\right)\right]}^{-1}X^{T}W\left(u_{i},v_{i}\right)Y,$$

where $W\left(u_{i},v_{i}\right)$ is the square matrix of weights relative to the position of $\left(u_{i},v_{i}\right)$ in the study area, $X^{T}W\left(u_{i},v_{i}\right)X$ is the geographically weighted variance–covariance matrix, and Y is the vector of the values of the dependent variable.

The weighted matrix $W\left(u_{i},v_{i}\right)$ for geographic location i, in its leading diagonal, is calculated as follows [39]:

(3) $$W\left(u_{i},v_{i}\right)=\left[\begin{array}{ccc} w_{1}\left(u_{i},v_{i}\right) & o & o\\ o & \ldots & o\\ o & o & w_{n}\left(u_{i},v_{i}\right) \end{array}\right].$$

Within the matrix, $W_{n}\left(u_{i},v_{i}\right)$ refers to the weight assigned to location i. The GWR model can assess spatial influences among neighborhoods, which cannot be achieved through an OLS model. This means that data points closer to each other are more influential in the local regression than data points distant from each other. The weight was calculated by applying a Gaussian function, which is frequently used for applications with regular spatial distributions [40].

Moran’s I values were used to determine the spatial autocorrelation of the residuals in our spatial models. Moran’s I value approaches 0, spatial autocorrelation rarely appears and the residual values were randomly dispersed; as it approaches +1, a positive spatial autocorrelation exists; and as it approaches −1, a negative spatial autocorrelation exists [41].

## Figures and Tables

**Table 1:** Comparison between ordinary least squares (OLS) and geographically weighted regression (GWR) model results.

Category			OLS			GWR		
			Coefficient	SE	*t*-Value ^a^	2.5%	50%	97.5%
*A. pilosus*	Coefficient	Intercept	0.000	0.059	0.000	−0.839	−0.141	0.894
		Residential area	0.492	0.059	8.376 **	0.134	0.416	0.602
		Bio7	0.421	0.059	7.171 **	−0.310	0.333	0.704
	Performance	Adjusted *R*^2^	0.44			0.66		
		AIC	379.8			304.2		
		Moran’s I for residuals	0.098 (*p* = 0.110)			0.078 (*p* = 0.078)		
*L. scariola*	Coefficient	Intercept	0.000	0.067	0.000	−0.365	0.030	0.335
		Residential area	0.392	0.085	4.604 **	−0.133	0.279	0.675
		Mountain	−0.186	0.085	−2.186 *	−0.486	−0.278	−0.013
	Performance	Adjusted *R*^2^	0.27			0.48		
		AIC	422.1			379.6		
		Moran’s I for residuals	0.121 (*p* = 0.023)			0.047 (*p* = 0.184)		

^a^ Significance level: ** *p* < 0.001, * *p* < 0.05; bio7: annual temperature range; AIC: Akaike Information Criterion.

**Table 2:** Descriptive statistics of *A. pilosus* and *L. scariola* population density and 12 explanatory variables in 165 districts.

Category	Variables	Mean	Minimum	Maximum	SD	Moran’s I
Dependent variables	*A. pilosus* population/100 km^2^	3.2	0	32.8	5.3	0.632 *
	*L. scariola* population/100 km^2^	2	0	25.7	3.4	0.316 *
Anthropogenic activity	Population (people/km^2^)	1101	19	16,074	2457	0.571 *
	Residential area (%)	4.9	0.04	53.7	8.4	0.505 *
	Industrial area (%)	2.4	0.02	20.1	3.7	0.338 *
	Road (km/km^2^)	1.6	0.3	13.7	1.9	0.397 *
Land use	Stream area (%)	3	0.002	13.1	2.1	0.285 *
	Farm (%)	20.1	2.6	50	9.9	0.607 *
	Mountain (%)	58.5	15.2	89.4	17.7	0.664 *
Topographic properties	Elevation (m)	175.3	7.0	894	158.3	0.686 *
	Normalized TWI	0.42	0	1	0.22	0.613 *
Climate properties	Bio1 (°C)	11.6	7.9	14.1	1.3	0.836 *
	Bio7 (°C)	35.3	27.3	38.4	2.1	0.847 *
	Bio12 (mm)	1296	1100	1869	104	0.869 *

TWI: topographic wetness index, bio1: annual mean temperature, bio7: annual temperature range, bio12: annual precipitation. * Statistically significant Moran’s I value (*p* < 0.001).

## Data Availability

Not applicable.

## Supplementary Materials

The following are available online at https://www.mdpi.com/article/10.3390/plants10071377/s1, Figure S1: Pearson correlation matrix comparing paired variables. Figure S2: Raw values of transformed environmental variables.
