# Supplementary material for: Spatially Varying Relationships between Alien Plant Distributions and Environmental Factors in South Korea
Source: Plants (Basel). 2021 Jul 5;10(7):1377. doi: 10.3390/plants10071377 (PMC8309232; doi:10.3390/plants10071377)

Supplementary Materials

Figure S1. Pearson correlation matrix comparing paired variables. Positive correlations are shown in blue and negative correlations are in red.

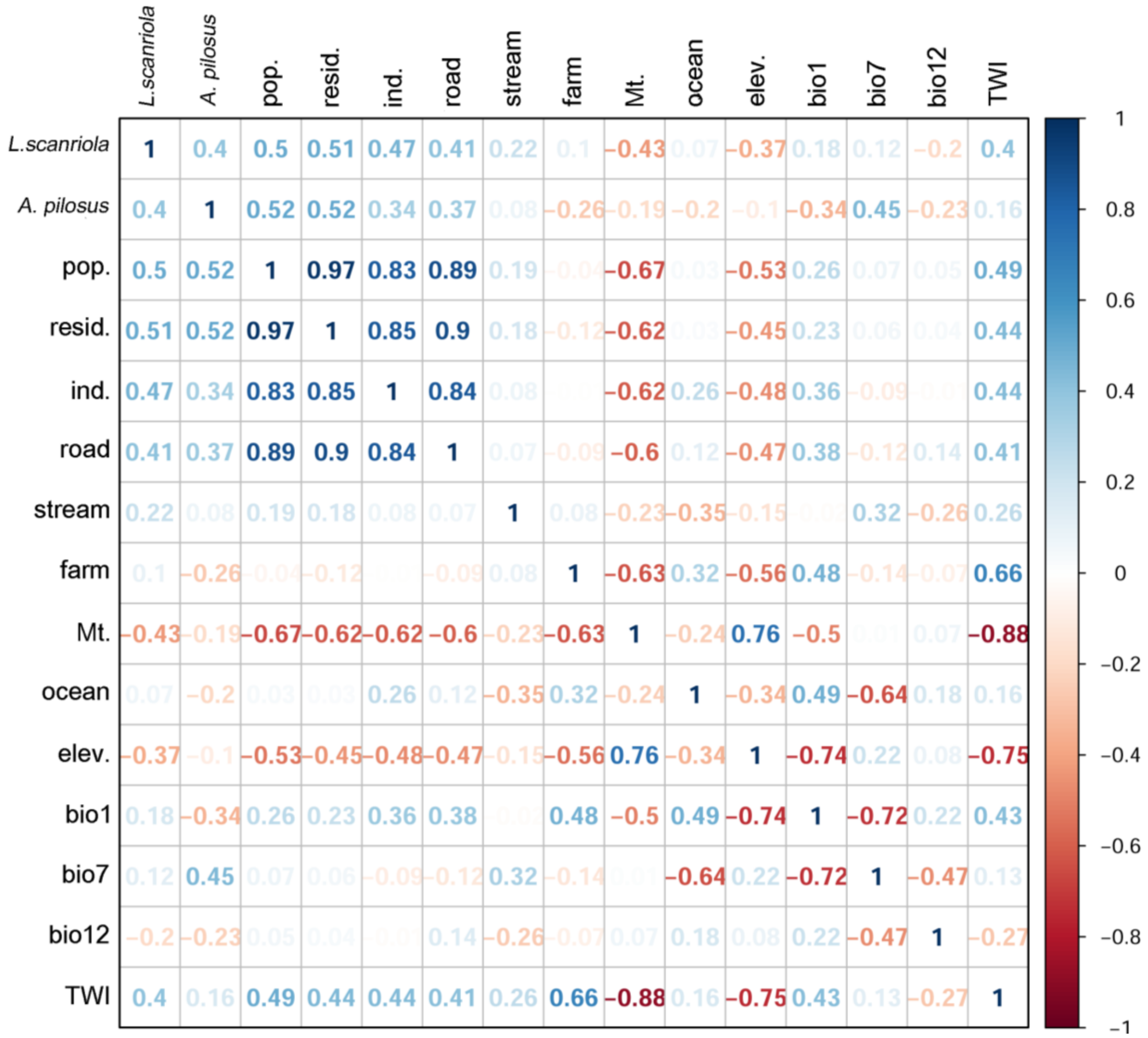

Figure S2. Maps of raw values of transformed environmental variables

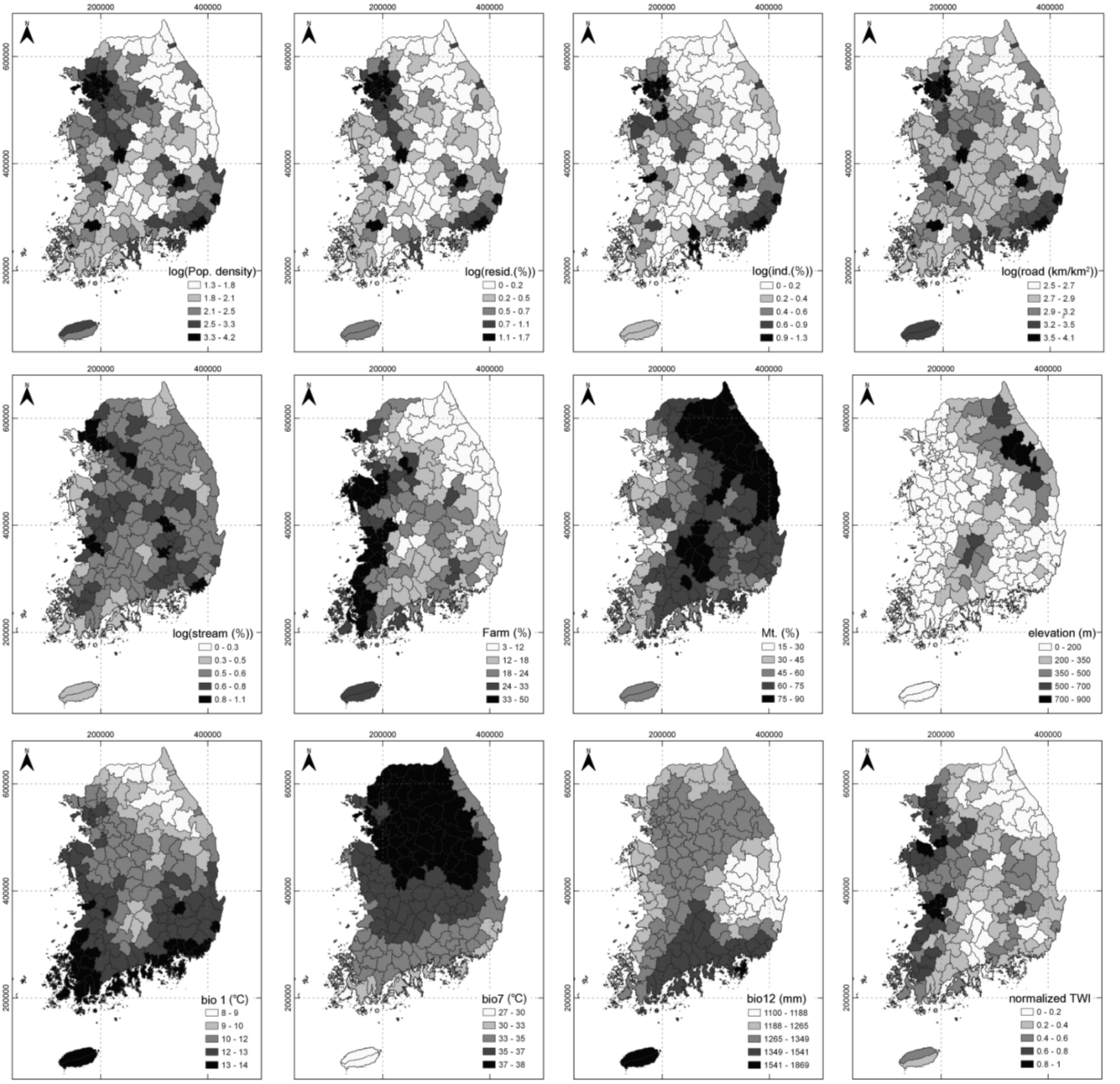

Supplement: Supplementary file 1 [file plants-10-01377-s001.zip › plants-1251163-supplementary.pdf]
